# Thresholds and timing of pre-operative thrombocytosis and ovarian cancer survival: analysis of laboratory measures from electronic medical records

**DOI:** 10.1186/s12885-016-2660-z

**Published:** 2016-08-08

**Authors:** Gabriella D. Cozzi, Jacob M. Samuel, Jason T. Fromal, Spencer Keene, Marta A. Crispens, Dineo Khabele, Alicia Beeghly-Fadiel

**Affiliations:** 1 Division of Epidemiology, Department of Medicine, Vanderbilt University Medical Center, 2525 West End Avenue, 838-A, Nashville, TN 37203 USA; 2 Division of Gynecologic Oncology, Department of Obstetics and Gynecology, Vanderbilt University Medical Center, Nashville, TN 37203 USA; 3 Vanderbilt-Ingram Cancer Center, Nashville, TN 37203 USA

**Keywords:** Platelets, Thrombocytosis, Ovarian cancer, Survival, Electronic medical records

## Abstract

**Background:**

Thrombocytosis has been associated with poor ovarian cancer prognosis. However, comparisons of thresholds to define thrombocytosis and evaluation of relevant timing of platelet measurement has not been previously conducted.

**Methods:**

We selected Tumor Registry confirmed ovarian, primary peritoneal, and fallopian tube cancer cases diagnosed between 1995–2013 from the Vanderbilt University Medical Center. Laboratory measured platelet values from electronic medical records (EMR) were used to determine thrombocytosis at three thresholds: a platelet count greater than 350, 400, or 450 × 10^9^/liter. Timing was evaluated with 5 intervals: on the date of diagnosis, and up to 1, 2, 4, and 8 weeks prior to the date of diagnosis. Cox regression was used to calculate hazard ratios (HR) and confidence intervals (CI) for association with overall survival; adjustment included age, stage, grade, and histologic subtype of disease.

**Results:**

Pre-diagnosis platelet measures were available for 136, 241, 280, 297, and 304 cases in the five intervals. The prevalence of thrombocytosis decreased with increasing thresholds and was generally consistent across the five time intervals, ranging from 44.8–53.2 %, 31.6–39.4 %, and 19.9–26.1 % across the three thresholds. Associations with higher grade and stage of disease gained significance as the threshold increased. With the exception of the lowest threshold on the date of diagnosis (HR_350_: 1.55, 95 % CI: 0.97–2.47), all other survival associations were significant, with the highest reaching twice the risk of death for thrombocytosis on the date of diagnosis (HR_400_: 2.01, 95 % CI: 1.25–3.23).

**Conclusions:**

Our EMR approach yielded associations comparable to published findings from medical record abstraction approaches. In addition, our results indicate that lower thrombocytosis thresholds and platelet measures up to 8 weeks before diagnosis may inform ovarian cancer characteristics and prognosis.

## Background

Ovarian cancer is a rapidly progressive and lethal disease. In the United States (US), 22,280 new cases and 14,240 deaths due to ovarian cancer are estimated to occur [1]. Ovarian cancer is the 5th leading cause of cancer deaths among women and is responsible for more deaths per year than any other gynecologic malignancy [1]. Because of the anatomic location within the peritoneal cavity, ovarian cancer may be very advanced or even distantly metastatic before a patient experiences symptoms. Further, these symptoms are often initially vague and non-specific, and may mimic a variety of benign conditions [2]. Ovarian cancer also lacks a detectable pre-invasive stage that can be reliably evaluated by screening on a population level [2]. As a result, over 60 % of ovarian cancer presents with advanced stage disease [1–3]. Recent US data indicate a dismal five year relative survival rate of 46 %; this is reduced to 28 % among cases with distant metastases [1].

The association between thrombocytosis and the presence of an underlying solid tumor has long been recognized, prompting investigation of the role of platelets in disease progression [4]. Platelets promote cancer cell survival through a variety of mechanisms, including protection from immune surveillance, promotion of angiogenesis, and arrest of the cancer cell cycle [5]. Platelets have also been shown to increase the proliferation rate of ovarian cancer cells indepedent of direct contact with those cells and unaffected by blockade of adhesion receptors [6]. Molecular studies have proffered a possible mechanism for thrombocytosis in advancing tumor growth. Tumor derived interleukin-6 increases hepatic thrombopoietin, which stimulates bone marrow megakaryocytes and platelet production of TGF-β1, which in turn activates the TGF-β1/smad proliferation pathway in tumor cells [7]. Additionally, in vitro knockdown of TGF-βR1 in ovarian cancer cells by an anti-TGF-βR1 antibody halts proliferation of cancer cells when exposed to platelets [6]. Using an orthotopic mouse model of ovarian cancer, platelet transfusion resulted in increased tumor growth, and platelets were demonstrated to protect cancer cells from apoptosis [8]. The persistent paracrine cycle in which platelets promote tumor cell proliferation and sustain cancer cell viability may underlie differences in cancer prognosis according to platelet count.

The majority of ovarian malignancies are epithelial, which has worse survival than other ovarian tumors [3]. Known prognostic factors for epithelial ovarian cancer include age, stage, grade histologic subtype, and optimal cytoreduction [7, 9, 10]. In addition, pre-diagnosis thrombocytosis has been associated with poor prognosis [7–9, 11–19]. To date, more than ten studies have evaluated the prognostic significance of preoperative thrombocytosis in ovarian cancer [7–9, 11–20]; all but one found that thrombocytosis was an independent negative factor in ovarian cancer survival [20]. However, the diagnostic threshold used to define thrombocytosis has varied from 300 to 450 × 10^9^/liter (L). Further, studies have used various time intervals for platelet measurements relative to diagnosis. Because of a lack of uniformity in thresholds and timing of platelet counts used to evaluate the association between thrombocytosis and overall survival in the existing literature, this study was undertaken to systematically compare three thresholds for thrombocytosis and the relevant timing of pre-diagnosis platelet counts in relation to ovarian cancer survival using Tumor Registry confirmed cases from the Vanderbilt University Medical Center (VUMC).

## Methods

### Study population

**Fig. 1:**
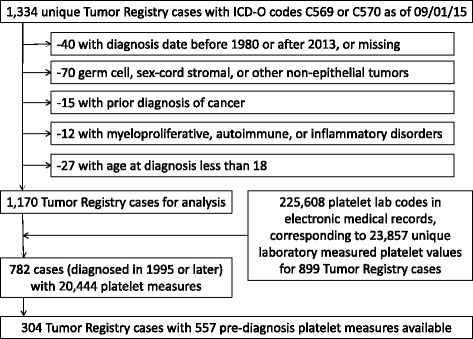
Flow Chart of Tumor Registry and Platelet Lab Data Preparation of de-identified Electronic Medical Records from the Vanderbilt University Medical Center

Appropriate Institutional Review Board (IRB) approval was garnered for this retrospective cohort study of de-identified EMR data (Vanderbilt University IRB #121299). Primary ovarian, peritoneal, and fallopian tube cancer cases were selected by International Classification of Disease-Oncology (ICD-O) codes C569 and C570 from the VUMC Tumor Registry (Fig. 1). Cases diagnosed before 1980, after 2013, or with unkown dates of diagnosis were excluded (*N =* 40). Germ cell tumors (ICD-O 9060, 9064, 9071, 9080, 9082, 9084, 9085), sex-cord stromal tumors (ICD-O 8620, 8634, 8640, 8670), and other tumors (ICD-O 8240, 8243, 8800, 8802, 8890, 8910, 9500, 9680) were excluded (*N =* 70). Epithelial ovarian cancer (EOC) cases were classified by histologic subtype: serous/papillary (ICD-O codes 8050, 8260, 8441, 8442, 8450, 8451, 8460, 8461, 8462); mucinous (ICD-O codes 8470, 8471, 8472, 8473, 8480, 8490); endometrioid (ICD-O codes 8380), clear cell (ICD-O codes 8310, 8313); and other (ICD-O codes 8013, 8041, 8046, 8070, 8120, 8320, 8570, 8950, 8951, 8980, 9000). Ovarian cancer cases with unknown histologic subtypes (ICD-O codes 8000, 8010, 8020, 8021, 8140, 8143, 8255, 8323, 8410, 8440, 8560) were retained, as the majority was likely to be epithelial. In addition to primary tumor site and histologic subtype, Tumor Registry data included date of diagnosis, stage, and grade of disease; women determined to have an age at diagnosis of less than 18 were excluded (*N =* 27). Women who had a prior epithelial or invasive carcinoma other than ovarian (*N =* 15), history of a myeloproliferative or myelodysplastic disorder (*N =* 2), or an autoimmune or inflammatory disorder (*N =* 10) were also excluded from analysis; no patients were found to have a history of total splenectomy (ICD code 41.5) prior to ovarian cancer diagnosis.

Laboratory values for pre-diagnosis platelet counts were selected from the Synthetic Derivative (SD), a de-identified mirror of electronic medical records (EMR) from VUMC. Platelet count measurements (Current Procedural Terminology (CPT) code 85049) were from Sysmex assays conducted on whole blood samples with a reference range of 135–370 × 10^9^/L by the Vanderbilt Pathology Lab Service. Thrombocytosis was defined using three thresholds: platelet counts greater than 350, 400, or 450 × 10^9^/L. The relevant time frame of platelet measurement was evaluated with 5 time intervals: on the date of diagnosis, and up to 1 week, 2 weeks, 4 weeks, 8 weeks before and including the date of diagnosis. Only pre-diagnosis platelet counts were analyzed, as paraneoplastic mechanisms are thought to drive thrombocytsosis [7]. Further, post-operative platelet measures are intrinsically altered by inflammation secondary to surgical stress, and can be iatrogenically changed by transfusion or blood loss during debulking surgery for ovarian cancer [21, 22]. Death from any cause was determined from EMR and by linkage to the National Death Index (NDI). Cases were considered to have died if they were listed as deceased in the SD or if there was a date of death from the NDI. Otherwise, overall survival was censored at the date of last EMR entry.

### Statistical analysis

Differences in clinical and histologic characteristics between cases with and without thrombocytosis were examined with Student’s *t* tests, χ^2^ tests, and Fisher’s exact test as appropriate. Cox proportional hazards regression was used to derive hazard ratios (HRs) and 95 % confidence intervals (CIs) for associations between thrombocytosis and overall ovarian cancer survival. Calendar time was used as the time scale for Cox regression models, with entry at date of ovarian cancer diagnosis and exit at date of death or last EMR entry. Due to low numbers, survival times were truncated at 10 years to prevent unstable estimates. Regression models included adjustment for known prognostic factors, including age at diagnosis, stage, grade, and histologic subtype of disease. Survival functions were visualized with Kaplan-Meier plots; the log-rank test was used to assess if differences were significant. Manual review of EMR was conducted to validate the date of diagnosis and timing of platelet measurement for a subset of cases. Sensitivity analyses were conducted by excluding cases with low malignant potential (LMP) tumors, synchronous cancers, non-White cases, and those with unknown stage of disease or histologic subtype. Data preparation was conducted with Excel and Python. In Python, the csv, datetime, time, matplotlib, and numpy modules were used along with dictionary and list stat structures to sort and filter data by platelet count and date relative to diagnosis date. All analyses were performed using SAS version 9.4 (SAS Institute, Cary, NC). A two-sided probability of 0.05 was used to determine statistical significance.

## Results

Table 1 presents the demographic and histopathologic characteristics of 1,170 Tumor Registry confirmed cases diagnosed between 1980 and 2013, and 304 cases with pre-diagnostic platelet measures available from the VUMC SD. No cases diagnosed before 1995 were found to have laboratory measured platelet values available in their EMR. Although age at diagnosis, primary site, and histologic subtype were generally comparable, fewer cases with platelet count measures available had either unknown or unstaged disease (8.22 vs. 27.78 %) or unknown grade (27.63 vs. 38.97 %) than all cases. Among cases with pre-diagnosis platelet measures available, the majority were white (*N =* 266, 87.5 %), and had advanced stage (III or IV, *N =* 191, 62.83 %), high grade (poorly differentiated or undifferentiated, *N =* 155, 51.0 %), and serous histologic subtypes (*N =* 147, 48.4 %) of disease.

**Table 1:** Clinical Characteristics of Tumor Registry Confirmed Ovarian Cancer Cases from the Vanderbilt University Medical Center

	All Epitheilal or Unknown Cases (*N* = 1170)		Cases With Pre-Diagnosis Platelets Measured (*N* = 304)	
Characteristic	N or mean	% or std dev^a^	N or mean	% or std dev^a^
Age at Diagnosis, years	58.5	13.7	60.2	14.5
Date of Diagnosis, calendar year				
1980–1984	117	10.0		
1985–1989	143	12.2		
1990–1994	183	15.6		
1995–1999	202	17.3	62	20.4
2000–2004	199	17.0	83	27.3
2005–2009	208	17.8	100	32.9
2010–2013	118	10.1	59	19.4
Race				
White	1036	88.6	266	87.5
Black	68	5.8	25	8.2
Asian	11	0.9	2	0.7
Native	2	0.2	0	0.0
Other/Unknown	53	4.5	11	3.6
Primary Site				
Ovary (C569)	1129	96.5	294	96.7
Fallopian Tube (C570)	41	3.5	10	3.3
Histologic Subtype				
Serous	666	56.9	147	48.4
Endometrioid	105	9.0	33	10.9
Mucinous	75	6.4	24	7.9
Clear Cell	45	3.9	15	4.9
Other	47	4.0	18	5.9
Unknown	232	19.8	67	22.0
Stage of Disease				
I	172	14.7	69	22.7
II	52	4.4	19	6.3
III	360	30.8	124	40.8
IV	261	22.3	67	22.0
Unknown/Unstaged	325	27.8	25	8.2
Disease Grade				
1, Well differentiated	58	5.0	21	6.9
2, Moderately differentiated	161	13.8	44	14.5
3, Poorly differentiated	412	35.2	120	39.5
4, Undifferentiated	83	7.1	35	11.5
Unknown	456	39.0	84	27.6
Overall Survival, years	4.1	3.4	3.4	3.1

^a^Percentages may not sum to 100 due to rounding error

Associations between thrombocytosis and clinical covariates are summarized in Table 2. When using the lowest thrombocytosis threshold, associations with higher stage (*P*-value =0.051) and grade (*P*-value =0.115) were suggestive, but not significant. Using either of the higher thresholds, cases were more likely to have stage IV or unstaged disease (*P*-value_400_ = 0.038, and *P*-value_450_ = 0.009) and undifferentiated grade 4 tumors (*P*-value_400_ = 0.008, and *P*-value_450_ = 0.010) than cases without thrombocytosis. A significant association was seen with primary site at the middle threshold (*P*-value_400_ = 0.015), but this did not evident at either of the other thresholds. Regardless of threshold used, there was no association between thrombocytosis and age, race, or histologic subtype of disease.

**Table 2:** Associations Between Thrombocytosis within 8 Weeks of Diagnosis and Clinical Covariates among Ovarian Cancer Cases from the Vanderbilt University Medical Center

	Thrombocytosis (>350 × 10^9^/L)					Thrombocytosis (>400 × 10^9^/L)					Thrombocytosis (>450 × 10^9^/L)				
	No (*N* = 145)		Yes (*N* = 159)			No (*N* = 190)		Yes (*N* = 114)			No (*N* = 228)		Yes (*N* = 76)		
Characteristic	N or mean		(% or std dev)^a^		*P*-value**	N or mean		(% or std dev)^a^		*P*-value**	N or mean		(% or std dev)^a^		*P*-value**
Age at Diagnosis, years	60.7	(14.1)	59.9	(14.8)	0.631	60.9	(14.3)	59.1	(14.8)	0.275	60.4	(14.4)	59.8	(14.8)	0.746
Race															
White	128	(88.3)	138	(86.8)	0.696	169	(89.0)	97	(85.1)	0.325	202	(88.6)	64	(84.2)	0.317
Other/Unknown	17	(11.7)	21	(13.2)		21	(11.1)	17	(14.9)		26	(11.4)	12	(15.8)	
Primary Site															
Ovary (C569)	137	(94.5)	157	(98.7)	0.052 †	114	(100.0)	114	(100.0)	**0.015 †**	218	(95.6)	76	(100.0)	0.072 †
Fallopian Tube (C570)	17	(5.5)	2	(1.3)		0	(0.0)	0	(0.0)		10	(4.4)	0	(0.0)	
Histologic Subtype															
Serous	68	(46.9)	79	(49.7)	0.691	88	(46.3)	59	(51.8)	0.629	111	(48.7)	36	(47.4)	0.414
Endometrioid	14	(9.7)	19	(12.0)		20	(10.5)	13	(11.4)		24	(10.5)	9	(11.8)	
Mucinous	15	(10.3)	9	(5.7)		19	(10.0)	5	(4.4)		22	(9.7)	2	(2.6)	
Clear Cell	6	(4.1)	9	(5.7)		9	(4.7)	6	(5.3)		10	(4.4)	5	(6.6)	
Other	9	(6.2)	9	(5.7)		11	(5.8)	7	(6.1)		12	(5.3)	6	(7.9)	
Unknown	33	(22.8)	34	(21.4)		43	(22.6)	24	(21.1)		49	(21.5)	18	(23.7)	
Serous	68	(46.9)	79	(49.7)	0.888	88	(46.3)	59	(51.8)	0.646	111	(48.7)	36	(47.4)	0.923
Non-Serous	44	(30.3)	46	(28.9)		59	(31.1)	31	(27.2)		68	(29.8)	22	(29.0)	
Unknown	33	(22.8)	34	(21.4)		43	(22.6)	24	(21.1)		49	(21.5)	18	(23.7)	
Stage of Disease															
I	43	(29.7)	26	(16.4)	0.051	52	(27.4)	17	(14.9)	**0.038**	59	(25.9)	10	(13.2)	**0.009**
II	10	(6.9)	9	(5.7)		14	(7.4)	5	(4.4)		17	(7.5)	2	(2.6)	
III	56	(38.6)	68	(42.8)		76	(40.0)	48	(42.1)		94	(41.2)	30	(39.5)	
IV	25	(17.2)	42	(26.4)		35	(18.4)	32	(28.1)		43	(18.9)	24	(31.6)	
Unknown/Unstaged	11	(7.6)	14	(8.8)		13	(6.8)	12	(10.5)		15	(6.6)	10	(13.2)	
Disease Grade															
1, Well differentiated	10	(6.9)	11	(6.9)	0.115	15	(7.9)	6	(5.3)	**0.008**	18	(7.9)	3	(4.0)	**0.010**
2, Moderately differentiated	21	(14.5)	23	(14.5)		29	(15.3)	15	(13.2)		36	(15.8)	8	(10.5)	
3, Poorly differentiated	57	(39.3)	63	(39.6)		78	(41.1)	42	(36.8)		90	(39.5)	30	(39.5)	
4, Undifferentiated	10	(6.9)	25	(15.7)		12	(6.3)	23	(20.2)		18	(7.9)	17	(22.4)	
Unknown	47	(32.4)	37	(23.3)		56	(29.5)	28	(24.6)		66	(29.0)	18	(23.7)	

^a^Percentages may not sum to 100 % due to rounding error

**P-values from χ^2^ test or Fisher’s exact test where indicated (†); bold values denote significant associations

In Table 3, associations between thrombocytosis and overall ovarian cancer survival are shown, including 5 time intervals and three thresholds. Within each threshold, the prevalence of thrombocytosis was lowest on the date of diagnosis, but this was not found to significantly differ from the other timeframes. In both unadjusted and multivariable adjusted analysis, thrombocytosis was significantly associated with worse survival across all five time intervals and three thresholds, except for the lowest threshold on the date of diagnosis (*P*-value_350crude_ = 0.093 and *P*-value_350adjusted_ = 0.070). Other associations at this threshold indicated a 79-83 % significantly increased risk of death with thrombocytosis. For both of the higher thresholds, associations were larger on the date of diagnosis (HR_400_: 2.01, 95 % CI 1.25–3.23, and HR_450_: 2.00, 95 % CI: 1.16–3.46), and smaller as time to diagnosis increased up to 8 weeks (HR_400_: 1.55, 95 % CI: 1.14–2.10, and HR_450_: 1.55, 95 % CI: 1.12–2.15). Kaplan-Meier analysis was found to be in general agreement, such that ovarian cancer cases with pre-diagnostic thrombocytosis were found to have significantly shorter survival across the 400 threshold regardless of timing, and significantly shorter survival for the 350 and 450 thresholds for all time periods except for those taken on the date of diagnosis (data not shown).

**Table 3:** Thrombocytosis and Overall Survival Among Ovarian Cancer Cases from the Vanderbilt University Medical Center

	Thrombocytosis			No Thrombocytosis (Reference)		Unadjusted Association			Multivariable Association^a^		
Thrombocytosis	%	N Cases	N Events	N Cases	N Events	HR	95 % CI	*P*-value*	HR	95 % CI	*P*-value*
Defined by ≥350 × 10^9^/L											
Date of diagnosis	44.8	61	42	75	46	1.45	0.94–2.23	0.093	1.55	0.97–2.47	0.070
1 week prior to date of diagnosis	53.1	128	96	113	63	**2.09**	**1.51–2.89**	**<0.001**	**1.83**	**1.30–2.58**	**0.001**
2 week prior to date of diagnosis	52.5	147	106	133	72	**1.96**	**1.47–2.66**	**<0.001**	**1.79**	**1.31–2.44**	**<0.001**
4 week prior to date of diagnosis	53.2	158	110	139	73	**1.94**	**1.44–2.62**	**<0.001**	**1.80**	**1.33–2.45**	**<0.001**
8 week prior to date of diagnosis	52.3	159	111	145	77	**1.92**	**1.43–2.58**	**<0.001**	**1.80**	**1.33–2.43**	**<0.001**
Defined by ≥400 × 10^9^/L											
Date of diagnosis	31.6	43	33	93	60	**1.98**	**1.27–3.09**	**0.003**	**2.01**	**1.25–3.23**	**0.004**
1 week prior to date of diagnosis	39.4	95	73	146	94	**1.92**	**1.39–2.64**	**<0.001**	**1.62**	**1.16–2.26**	**0.005**
2 week prior to date of diagnosis	38.2	107	79	173	108	**1.87**	**1.39–2.53**	**<0.001**	**1.60**	**1.17–2.18**	**0.003**
4 week prior to date of diagnosis	38.1	113	80	184	112	**1.78**	**1.32–2.40**	**<0.001**	**1.54**	**1.14–2.09**	**0.006**
8 week prior to date of diagnosis	37.5	114	81	190	116	**1.78**	**1.33–2.40**	**<0.001**	**1.55**	**1.14–2.10**	**0.005**
Defined by ≥450 × 10^9^/L											
Date of diagnosis	19.9	27	22	109	71	**1.98**	**1.21–3.27**	**0.007**	**2.00**	**1.16–3.46**	**0.013**
1 week prior to date of diagnosis	26.1	63	51	178	116	**2.01**	**1.43–2.82**	**<0.001**	**1.71**	**1.20–2.45**	**0.003**
2 week prior to date of diagnosis	25.7	72	55	208	132	**1.91**	**1.38–2.65**	**<0.001**	**1.62**	**1.16–2.27**	**0.005**
4 week prior to date of diagnosis	24.9	74	56	223	136	**1.88**	**1.37–2.59**	**<0.001**	**1.56**	**1.12–2.17**	**0.008**
8 week prior to date of diagnosis	25.0	76	57	228	140	**1.79**	**1.30–2.45**	**<0.001**	**1.55**	**1.12–2.15**	**0.009**

*Bold type denotes significant associations

^a^Adjusted for age at diagnosis, stage, grade, and histologic subtype

**Fig. 2:**
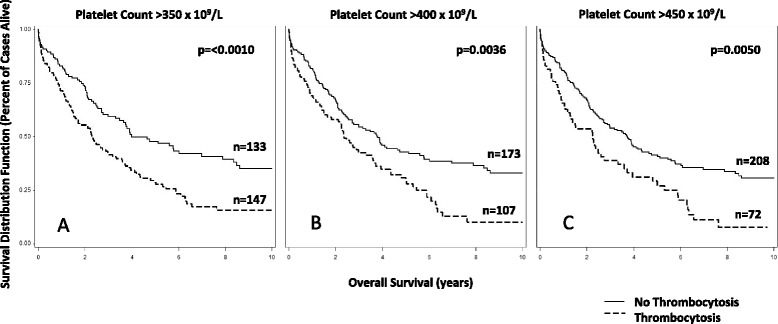
Ovarian Cancer Overall Survival Kaplan-Meier functions for Thrombocytosis within two weeks of diagnosis.; Legend: **a** Thrombocytosis (350) two weeks prior to and including the date of diagnosis; **b** Thrombocytosis (400) two weeks prior to and including the date of diagnosis; and **c** Thrombocytosis (450) two weeks prior to and including the date of diagnosis

To validate dates of platelet measurement and diagnosis, EMR were manually reviewed for twenty cases. The Tumor Registry date of diagnosis matched the date of operative and/or pathology report for 75 % of reviewed cases. However, for 25 %, the Tumor Registry date of diagnosis was up to 2 weeks before the operative and/or pathology report, and usually coincided with the first presentation of symptoms that led to the diagnosis, e.g., computed tomography (CT) or ultrasound imaging. Based on this, we selected 2 weeks prior to and including the date of diagnosis as the interval most likely to best capture pre-diagnosis thrombocytosis in our data, and used this to conduct sensitivity analyses (Table 4). In agreement with our primary analysis, excluding cases with low malignant potential (*N =* 12), synchronous cancers (*N =* 26), not reported as White (*N =* 38), or unknown stage of disease (*N =* 36), did not materially alter our results. When unknown histologic subtypes (*N =* 86) were excluded, significance was attenuated for two of the three thresholds (*P*-value_350_ = 0.034; *P*-value_400_ = 0.089; *P*-value_450_ = 0.186). When all above exclusions were simultaneously applied, all associations were attenuated (*P*-value_350_ = 0.065; *P*-value_400_ = 0.072; *P*-value_450_ = 0.096), likely due to the small sample size remaining in the analysis (*N =* 152). Similar to Cox regression, Kaplan-Meier plots showed significant differences for cases with and without thrombocytosis within two weeks of diagnosis for all three thresholds evaluated (Fig. 2).

**Table 4:** Sensitivity Analysis of Thrombocytosis within 2 Weeks of Diagnosis and Overall Ovarian Cancer Survival

	Thrombocytosis		No Thrombocytosis		Unadjusted Association			Multivariable Association^a^		
Thrombocytosis	N Cases	N Events	N Cases	N Events	HR	95 % CI	*P*-value*	HR	95 % CI	*P*-value*
Defined by ≥350 × 10^9^/L										
2 weeks to date of diagnosis	147	106	133	72	**1.96**	**1.45–2.66**	**<0.001**	**1.78**	**1.31–2.44**	**<0.001**
Excluding low malignant potential tumors	147	106	121	71	**1.71**	**1.26–2.32**	**<0.001**	**1.67**	**1.22–2.28**	**0.001**
Excluding synchronous cancers	134	101	124	71	**1.94**	**1.42–2.64**	**<0.001**	**1.72**	**1.26–2.36**	**<0.001**
Excluding non–Whites	128	91	114	61	**1.92**	**1.38–2.68**	**<0.001**	**1.88**	**1.34–2.62**	**<0.001**
Excluding unknown stage of disease	134	95	124	67	**1.89**	**1.37–2.60**	**<0.001**	**1.66**	**1.20–2.30**	**0.002**
Excluding unknown histologic subtypes	115	79	104	52	**1.81**	**1.27–2.58**	**0.001**	**1.48**	**1.03–2.13**	**0.034**
Excluding all of the above cases	84	60	68	40	1.47	0.98–2.20	0.063	1.47	0.98–2.21	0.065
Defined by ≥400 × 10^9^/L										
2 weeks to date of diagnosis	107	78	173	100	**1.87**	**1.39–2.53**	**<0.001**	**1.60**	**1.17–2.18**	**0.003**
Excluding low malignant potential tumors	107	78	161	99	**1.67**	**1.23–2.26**	**<0.001**	**1.50**	**1.10–2.06**	**0.010**
Excluding synchronous cancers	96	74	162	98	**1.94**	**1.42–2.64**	**<0.001**	**1.61**	**1.17–2.20**	**0.003**
Excluding non–Whites	91	64	151	88	**1.75**	**1.26–2.43**	**<0.001**	**1.58**	**1.13–2.21**	**0.008**
Excluding unknown stage of disease	96	69	162	93	**1.84**	**1.34–2.52**	**<0.001**	**1.49**	**1.08–2.07**	**0.016**
Excluding unknown histologic subtypes	84	59	135	72	**1.81**	**1.28–2.57**	**<0.001**	1.37	0.95–1.97	0.089
Excluding all of the above cases	59	44	93	56	**1.69**	**1.13–2.53**	**0.011**	1.46	0.97–2.20	0.072
Defined by ≥450 × 10^9^/L										
2 weeks to date of diagnosis	72	55	208	123	**1.91**	**1.38–2.65**	**<0.001**	**1.62**	**1.16–2.27**	**0.005**
Excluding low malignant potential tumors	72	55	196	122	**1.73**	**1.25–2.39**	**<0.001**	**1.53**	**1.09–2.14**	**0.014**
Excluding synchronous cancers	64	51	194	121	**1.92**	**1.38–2.68**	**<0.001**	**1.66**	**1.17–2.34**	**0.004**
Excluding non–Whites	60	44	182	108	**1.82**	**1.27–2.61**	**0.001**	**1.65**	**1.14–2.40**	**0.008**
Excluding unknown stage of disease	63	47	195	115	**1.88**	**1.33–2.65**	**<0.001**	**1.51**	**1.05–2.15**	**0.024**
Excluding unknown histologic subtypes	54	41	165	90	**1.81**	**1.24–2.63**	**0.002**	1.31	0.88–1.94	0.186
Excluding all of the above cases	37	29	115	71	**1.73**	**1.11–2.70**	**0.016**	1.49	0.93–2.37	0.096

*Bold type denotes significant associations

^a^Adjusted for age at diagnosis, stage, grade, and histologic subtype as appropriate after exclusions

## Discussion

In this large retrospective analysis of confirmed Tumor Registry cases from a single tertiary-care medical center with platelet measurments available in electronic medical records (EMR), we found that thrombocytosis, defined as a platelet count greater than 350, 400, or 450 × 10^9^/L, and measured up to 8 weeks before diagnosis, was associated with significantly shorter overall ovarian cancer survival. This work adds to existing literature by providing a comprehensive comparison of thrombocytosis prevalence and associations with survival across three thresholds with uniform platelet measures in one study population, and indicates that elevated platelet counts up to eight weeks before diagnosis may be informative for ovarian cancer prognosis. In addition, this research demonstrates the power of combining Tumor Registry and EMR data to evaluate potential prognostic factors as an alternate approach to analysis based on data from retrospective medical chart review.

To date, more than ten studies have analyzed pre-operative thrombocytosis in relation to ovarian cancer survival [7–9, 11–17, 19, 20]. The threshold to define thrombocytosis has varied from 300–450, with most studies using 400 [11–15, 17–20]. The relevant timing of thrombocytosis has also varied from within 1 week [12, 13, 18], to within 2 weeks from the date of diagnosis [14, 15, 19], although several publications lack specific details on timing other than qualifying measures as preoperative [8, 9, 11, 16, 20]. The prevalence of thrombocytosis has varied across studies, with ranges of 22.4 % [14] to 43.5 % [19]. Among those with significant associations with overall ovarian cancer survival, hazards have also ranged considerably. The largest association reported was a nearly five-fold increased risk of death among 136 cases, where adjustment included age, stage, histologic subtype, grade, cytoreduction, chemotherapy, CA-125, and fibrinogen [18]. In that study, the thrombocytosis threshold differs between the text (400) and table of results (300), but the reported prevalence was low (7.4 %) [18]. The largest study to date included 816 cases and had a prevalence of 22.8 % with a threshold of 400 [11]. After adjusting for age, menopausal status, histologic subtype, grade, stage, residual disease, ascites, CA-125, hemoglobin, and leukocyte count, cases with thrombocytosis had a more than two-fold higher risk of death [11]. The highest prevalence reported (42.9 %) used a threshold of 350 among 91 cases; after adjustment for age, menopausal status, stage, CA-125, and surgery, thrombocytosis was associated with a more than two-fold increased risk of death [16]. Meta-analysis of 5 studies with different statistical adjustments, but all using 400 as their threshold, yielded a thrombocytosis prevalence of 31.1 %, and a 52 % significantly increased risk of 5 year mortality [23]. Thus, our findings for the prevalence of pre-diagnosis thrombocytosis and associations with overall survival are in general agreement with existing literature, indicating that evaluation of VUMC Tumor Registry confirmed cases is a viable approach for ovarian cancer research.

Pre-operative thrombocytosis has been linked to decreased overall survival and to a host of clinical parameters and outcomes, which may in turn help elucidate the factors at play in increased mortality for those patients with thrombocytosis. Platelets are an acute phase reactant, meaning that platelet count transiently increases in response to inflammation [24]. Etiologies for reactive thrombocytosis include malignancy, tissue damage, infection, and chronic inflammation [22]. Factors influencing platelet count, in the absence of the the aforementioned reactive factors, include age, sex, race/ethnicity, nutritional status, drug exposure [24, 25], and inherent genetic variability [26]. Thrombocytosis in patients with ovarian cancer is associated with greater volumes of ascites [6, 13, 18], lower hemoglobin, receipt of peri-operative packed red blood cells transfusion [6], major post-operative complications [27] and post-operative death [25]. Thrombocytosis with concurrent leukocytosis, another marker of inflammation or infection, was associated with a higher risk of post-operative death (OR 5.4) than either thrombocytosis (OR 2.16) or leukocytosis alone (OR 1.78) [27]. Furthermore, pre-operative thrombocytosis was an independent negative prognostic factor for disease recurrence and progression-free survival [5, 6, 9, 12, 15–17, 19]. Taken together, these factors may explain, at least in part, the association between thrombocytosis and shorter overall survival.

In addition to overall mortality, pre-operative thrombocytosis was found to be an independent predictor for the development of venous thromboembolism among clear cell ovarian carcinoma cases [28]. Risk factors for venous thromboembolism in ovarian cancer include increasing age, a higher number of chronic comorbid conditions, higher stage disease, invasive histology, and the absence of any major surgery [29]. In a multivariable model, women with symptomatic venous thromboembolism at the time of diagnosis prior to primary treatment had significantly shorter overall survival when compared with women without venous thromboembolism at diagnosis [30]. In accordance with the guidelines provided by the American Society of Clinical Oncology, oncology patients should receive venous thromboembolism prophylaxis 7–10 days prior to a major operation and extending up to 4 weeks post-operatively in patients with abdominal or pelvic surgery with high risk features [31]. As our data indicate that thrombocytosis up to 8 weeks pre-operative may confer worse survival, and thrombocytosis has been linked to development of venous thromboembolism, more investigation is needed to determine the possible efficacy of longer periods of pre-operative thromboembolism prophylaxis in helping to reduce ovarian cancer morbidity and mortality. Robust findings from our sensitivity analysis results indicate that pre-operative prophylaxis may have clinical utility for all types of ovarian cancer cases.

This study expands upon existing knowledge by directly comparing three thresholds for thrombocytosis. As expected, the prevalence of thrombocytosis decreased with increasing threshold. For both the 400 and 450 thresholds, we found significant associations with higher stage and grade of disease, and significant associations with overall survival even after adjusting for clinical and tumor characteristics. Our study also expands upon current knowledge by analyzing multiple time widows for the occurrence of thrombocytosis. For each threshold, the prevalence of thrombocytosis was lowest on the date of diagnosis, and was fairly consistent across the remaining time frames. With regard to survival associations, when using the smallest threshold of 350, the association on the date of diagnosis was not significant, but increasing time frames all had significant associations with worse survival. This pattern differed for both of the larger thresholds, where stronger associations occurred on the date of diagnosis, and smaller, but still significant associations, were found when measures up to 8 weeks pre-diagnosis were included. Larger associations closer to the date of diagnosis may be due to disease progression and subsequent worsening paraneoplastic thrombocytosis. Alternately, inclusion of values up to 8 weeks pre-diagnosis may increase measurement error, such that cases with thrombocytosis initially evaluated for a separate indication may have resolution of thrombocytosis by the time of cancer diagnosis. Such misclassification of a dichotomous exposure would be independent of the outcome and would therefore serve to attenuate associations toward the null. Notably, our validity sub-study indicated that all platelet measures were from before diagnosis. In addition, we found that among cases with thrombocytosis occurring within 8 weeks of diagnosis, more than 95 % also had thrombocytosis within 4 weeks, 2 weeks, or 1 week of diagnosis, regardless of the threshold. Only when evaluating the smallest timeframe, the date of diagnosis, was this reduced to 70 % of cases. Thus, misclassification of thrombocytosis is not likely to greatly influence the results of the current study.

Limitations of this investigation include the number of cases with preoperative platelet count data available, when compared with all confirmed Tumor Registry cases. Differences in disease presentation, preoperative course, and time to diagnosis likely contribute to variability in whether platelets were measured before or after diagnosis for each patient. Data on use of anti-platelet or anti-coagulant medications, which may alter the potential for adverse events in the setting of thrombocytosis, was not included in this study. Further, many cases were missing information on histologic subtype, stage, or grade of disease. Sensitivity analyses conducted by excluding these cases still yielded mostly significant survival associations, indicating that missing data is not greatly impacting our findings. Another limitation is the lack of data on optimal tumor cytoreduction, which has been shown to be an important negative predictor of ovarian cancer survival [10]. However, thrombocytosis has not been found to be associated with optimal debulking in prior studies, so this should not confound the current results. While sucessess of surgical debulking was unavailable from Tumor Registry data, all included cases were reviewed by trained Tumor Registry personnel, and information on stage, grade, and subtype of disease were reasonably standardized. Additional limitations of this analysis include the inherent retrospective nature of our analysis, the possibility of including deaths unrelated to ovarian cancer by use of all-cause mortality, and care limited to a single tertiary care center. Thus, our findings may not be generalizable to all ovarian cancer cases at all institutions. However, outcomes were ascertained by linkage to the NDI as well as by EMR notes on vital status, and our results are generally in agreement with those from other single and multi-center studies. Additional strengths of the current study include a robust analytic approach that included three thrombocytosis thresholds, exploration of relevant time frames for platelet measurement, and multivariable adjustment for all clinical covariates available. In addition, our study employed compuater programming methods to obtain EMR data, which differs from other studies in which manual chart review was conducted. Rather than including cases without evidence of thrombocytosis noted in medical records, our reference group included only cases where platelet values were actually measured, and were not found to be elevated.

## Conclusions

Thrombocytosis was identified in 20-50 % of ovarian cancer cases, depending upon the pre-diagnostic time interval and diagnostic threshold. Regardless of timing or threshold, thrombocytosis was generally associated with more aggressive tumor characteristics and was an independent negative prognostic factor for overall survival. Our findings indicate that lower thrombocytosis thresholds and measures collected up to 8 weeks before diagnosis may inform ovarian cancer prognosis.

## Abbreviations

CI, confidence interval; CPT, Current Procedural Terminology (code); CT, computed tomography; EMR, electronic medical records; HR, hazard ratio; ICD-O, International Classification of Disease-Oncology; IRB, institutional review board; L, liter; NDI, National Death Index; SAS, Statistical Analysis Software; SD, Synthetic Derivative; TGF-β1, transforming growth factor-beta 1; TGF-βR1, transforming growth factor-beta receptor 1; US, United States; VUMC, Vanderbilt University Medical Center
